# The impact of reminiscent music therapy and robot-assisted rehabilitation on older stroke patients: a protocol for a randomized controlled trial

**DOI:** 10.3389/fneur.2024.1345629

**Published:** 2024-04-08

**Authors:** Qian Liu, Zuoyan Liu, Hong Cheng, Yang Xu, Fang Wang, Li Liu, Xiuying Hu

**Affiliations:** ^1^Innovation Center of Nursing Research, Nursing Key Laboratory of Sichuan Province, West China Hospital, and West China School of Nursing, Sichuan University, Chengdu, China; ^2^Department of Rehabilitation Medicine Center, West China Hospital, Sichuan University, Chengdu, China; ^3^West China School of Nursing, Sichuan University, Chengdu, China; ^4^School of Automation Engineering, University of Electronic Science and Technology of China, Chengdu, China

**Keywords:** music therapy, reminiscence, robot-assisted rehabilitation, stroke, older people

## Abstract

**Background:**

Stroke is the main disease that causes the burden of neurological disease, leading to upper limb dysfunction and affecting their self-care abilities. Robot-assisted rehabilitation therapy has been gradually used in the rehabilitation of upper limb function after stroke. However, it would be beneficial to explore auxiliary interventions such as reminiscent music therapy, a combination of music and reminiscent, to relieve negative emotions and post-stroke fatigue and improve rehabilitation outcomes. This protocol aims to evaluate the effectiveness of reminiscent music therapy combined with robot-assisted rehabilitation in older stroke patients.

**Methods:**

This trial is a single-blind, three-arm randomized controlled trial. Older stroke patients with upper limb dysfunction will be recruited. Participants will be randomly assigned to receive usual rehabilitation treatment and care, usual rehabilitation treatment and care plus robot-assisted rehabilitation and reminiscent music therapy, or usual rehabilitation treatment and care plus robot-assisted rehabilitation. Robot-assisted rehabilitation will be conducted by rehabilitation doctors five times per week for 3 weeks. In experimental group 1, a reminiscent song list will be played for patients. The primary outcome is activities of daily living. All outcomes will be evaluated at baseline and in the week immediately post-intervention.

**Discussion:**

We are conducting the first randomized controlled trial on the effects of reminiscent music therapy combined with robot-assisted rehabilitation in older stroke patients. It is expected that this study, if proven effective in improving the activities of daily living in older stroke patients with upper limb dysfunction, will provide evidence-based rehabilitation strategies for medical staff.

**Clinical Trial Registration**: ChiCTR2200063738.

## 1 Introduction

Stroke is the leading cause of death and disability in the world (1), and it is also the main disease that causes the burden of neurological disease in old people (2). Under the severe trend of population aging, the incidence rate of stroke increases exponentially with advancing years (3). Of the stroke patients in China, 50.81% are older people aged 60 years or older (4). Stroke is characterized by a high rate of disability, and most stroke patients will experience long-term neurological damage. Persistent upper limb disability occurs in up to 80% of stroke patients (5), which leads to the decline of self-care ability and the need to receive long-term rehabilitation training.

With the development of artificial intelligence technology, traditional medical equipment is constantly developing toward intelligence. Attracting attention in clinical practice, robot-assisted rehabilitation therapy has been gradually used in the rehabilitation of upper limb function after stroke. Some studies have suggested that, compared with traditional rehabilitation treatment, robot-assisted rehabilitation could significantly improve muscle coordination, upper limb function, and social participation in stroke patients (6–10). However, the care burden and economic pressure of patients will be aggravated when suffering from stroke, leading to negative emotions in patients, forming a negative coping style, and inducing post-stroke fatigue with an incidence of 25–85% (11–13). Post-stroke fatigue can affect the subjective feelings of older people’s independent activities and lead to exercise fatigue (14), contributing to a poor rehabilitation prognosis (15–17). In addition, stroke patients often have lower motor motivation, which may lead to a lack of rehabilitation motivation and hinder functional recovery (18). When patients have a high level of rehabilitation motivation, their participation also increases, which is beneficial for improving their activities of daily living (19). Therefore, it is necessary to add auxiliary intervention to alleviate the negative emotions of patients with upper limb dysfunction after stroke, so as to urge them to actively cope with functional rehabilitation treatment and finally improve rehabilitation outcomes.

Indicated to be physically and mentally helpful, reminiscence therapy has been gradually applied to improve the cognitive function and mental health of older patients with stroke (20–22). Music is one of the most common media to trigger nostalgia, which can not only have a positive effect on patients by evoking nostalgia but also treat physical or psychological diseases by musical sound and rhythm (23). A theoretical model suggested that music, when combined with nostalgia, can help summon autobiographical memories, evoke strong emotions, elicit physiological responses to improve physical wellbeing and immunity, and develop self-definition (24). Moreover, a previous study believed that adding music elements may increase the intrinsic motivation of patients in the process of rehabilitation treatment (25). Music activates both the auditory system and sensory-motor system simultaneously, and helps to reorganize and integrate information processing, executive control, and emotions, so as to shift attention away from fatigue and promote the acquisition of motor skills (26, 27). These literature provide a theoretical basis for the combination of reminiscent music therapy with robot-assisted rehabilitation. In accordance with these studies (20–22), it is suggested that the definition of reminiscent music therapy is a combination of music therapy and reminiscent therapy, meaning that using music can prompt and augment the recollection of autobiographical memories and help people think about the meaning of life and experiences, which helps to alleviate negative emotions and improve positive attitudes and hope levels.

Therefore, the aim of this study is to evaluate the efficacy of reminiscent music therapy combined with robot-assisted rehabilitation in improving the physical and mental health and promoting the self-care abilities of older stroke patients with upper limb dysfunction by conducting a randomized controlled trial. The hypothesis is that reminiscent music therapy combined with robot-assisted rehabilitation plus usual care may be more effective than robot-assisted rehabilitation plus usual care or only usual care with respect to improving activities of daily living and other outcomes.

## 2 Methods

**Figure 1:**
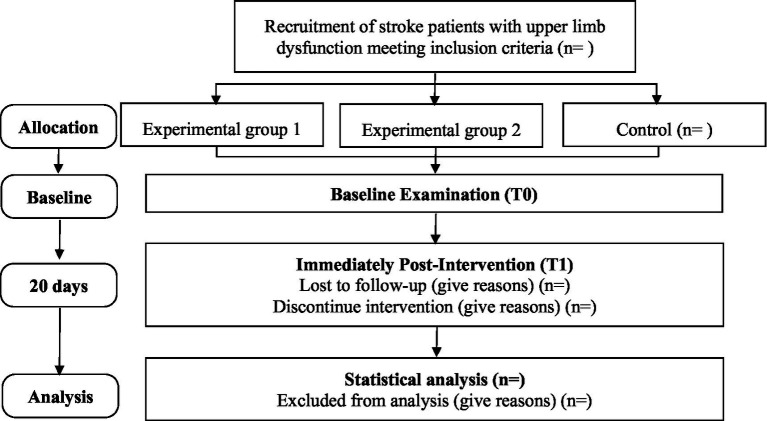
CONSORT flow diagram of this study.

This study is a single-blind, three-arm randomized controlled trial with randomization at the participant level. This trial was approved by the Ethics Committee of West China Hospital of Sichuan University (number: 2022–852) and was registered on the Chinese Clinical Trial Registry (number: ChiCTR2200063738). Activities of daily living will be the primary endpoint to examine whether usual rehabilitation treatment and care plus robot-assisted rehabilitation and reminiscent music therapy for older patients with upper limb dysfunction after stroke improve physical and mental health. All outcomes will be collected for three groups at baseline (T0) and in the week immediately post-intervention (T1). The overview of the study design is shown in Figure 1, and this study protocol followed the statement of the Consolidated Standard of Reporting Trials (CONSORT) (28).

### 2.1 Study setting

This trial will be conducted at the West China Hospital of Sichuan University in Chengdu, China. Stroke patients aged 60 years and older with upper limb dysfunction who are hospitalized in the Department of Neurology of this hospital will be recruited. The department is a national key clinical specialized department, which ensures that enough participants can be recruited for this trial. It is estimated that 10 stroke patients aged 60 and above can be accommodated per month, who are expected to be hospitalized for up to 20 days and can accept rehabilitation training for 3 weeks. Therefore, in accordance with the availability of the patients and a loss of follow-up rate of 10 to 15%, we plan to recruit 12 stroke patients aged 60 and above first to conduct a pilot study. The sample size will be calculated using PASS software based on the scores of activities of daily living after the pilot study. Each group will have no less than 35 people.

### 2.2 Recruitment

Participants will be recruited from the Department of Neurology at West China Hospital of Sichuan University. Researchers will recruit participants by approaching potentially eligible participants in the neurology ward. Inclusion criteria of participants are (1) newly admitted patients diagnosed as stroke, including ischemic or hemorrhagic strokes for the first time, (2) 60 years old and above, (3) conscious and in stable condition with stable vital signs, whether in the acute, subacute, or chronic phase of stroke, (4) with upper limb dysfunction and Brunnstrom stages I to VI, (5) normal cognitive function without minimal aphasia or hemineglect, (6) normal vision, hearing, and communication, (7) no music-related learning experience and acceptance of music, and (8) participating in this study voluntarily and signing the informed consent form. Exclusion criteria are (1) severe primary diseases of the heart, liver, kidney, and hematopoietic system, (2) a history of mental disease, (3) joint dislocation or skin damage of the upper limb, (4) largest slice diameter of cerebral infarction is greater than 3.0 cm, or the infarction spreads to two brain lobes, or the volume of intracerebral hematoma is greater than 30 mL, (5) uncooperative participants, (6) depression according to the Self-rating Depression Scale. After determining the eligibility of interested patients, the researchers will describe the details of this study to them again, obtain written consent forms, and collect baseline data.

### 2.3 Randomization

The random number table was generated based on the RV.Uniform function of SPSS 22.0 software. All participants will be randomly assigned to one of the three groups in a 1:1:1 ratio after initial evaluation. Participants will be randomly allocated via an online service provided by the Innovation Center of Nursing Research at the West China Hospital.

### 2.4 Blinding

Blinding of participants is not possible due to the obvious difference in intervention content. Participants of different groups would not live in the same ward, and the intervention would be implemented in a separate room. For practical reasons, researchers cannot be blinded because they need to clearly know the intervention process and content. In addition, follow-up of all participants, management, and other study tasks will be completed by the same researchers. A separation of tasks and blinding will be impossible. However, we will ensure the full concealment of allocation. The randomization of participants was conducted by a non-intervener by using sealed envelopes. Finally, the data collector and study statistician will be blinded to participants’ allocation.

### 2.5 Interventions

All participants will be randomly assigned to receive usual rehabilitation treatment and care (control), usual rehabilitation treatment and care plus robot-assisted rehabilitation and reminiscent music therapy (experimental group 1), or usual rehabilitation treatment and care plus robot-assisted rehabilitation (experimental group 2) in a 1:1:1 ratio after initial evaluation.

#### 2.5.1 Usual rehabilitation treatment and care

Usual rehabilitation treatment mainly includes drug treatment, comprehensive training of hemiplegic limbs, physical therapy, and so on, which will be formulated and implemented by rehabilitation doctors and neurologists according to the specific conditions of the participants. Usual rehabilitation care includes basic environmental care, diet care, drug care, health education, psychological care, and rehabilitation care, which will be formulated and implemented by specialized nurses according to the specific conditions of the participants.

#### 2.5.2 Robot-assisted rehabilitation

According to the specific conditions of the patients, the rehabilitation doctors will use the rehabilitation robot for the upper limb to carry out 40 min of rehabilitation training for the patients, five times per week for 3 weeks. The individualized rehabilitation scheme will be formulated by rehabilitation doctors and neurologists according to the specific situation of the patients. The robot equipment used in this study is a three-dimensional upper limb rehabilitation robot (model: A6) produced by Guangzhou Yikang Medical Equipment Industry Co., Ltd. Rehabilitation doctors need to undergo strict training from the manufacturer before they can operate the equipment. This robot equipment has five training modes: passive mode, active-passive mode, active mode, prescription mode, and track editing mode.

#### 2.5.3 Reminiscent music therapy

The same research group developed a music library of reminiscent songs in the early stage, including 100 Chinese songs released from 1935 to 1980, which are divided into 10 song lists and each list has 10 reminiscent songs. Each time the robot-assisted rehabilitation training of the upper limb is carried out, a reminiscent song list will be played for patients with a wireless headset. When playing music, the music beat and intensity will be between 45 and 60 decibels. After the robot-assisted rehabilitation training of the upper limb, the patients will be asked to sit down in a comfortable position and concentrate on breathing for 2 min. Patients should first slowly inhale for 3 s, pause for 1 s, then slowly exhale for 5 s, exhale all the residual gas in the lungs and imagine that the annoyance and unhappiness could be dissipated with the exhalation of the gas. The whole process of breathing relaxation is slow and uniform with rhythm, with a total of 5 cycles.

### 2.6 Outcomes and measurements

A research assistant who does not know the randomization scheme will perform the data collection and examinations at baseline (T0) and in the week immediately post-intervention (T1). Every time to measure data is face-to-face with participants. We expect the intervention to improve the abilities of patients to take care of themselves. Therefore, we choose the activities of daily living measured in the week immediately post-intervention (T1) as the primary outcome. Self-esteem, rehabilitation self-efficacy, positive emotion, upper limb function, and safety will be the secondary outcomes.

#### 2.6.1 Demographic data (T0)

To characterize the participants, the sex, age, educational level, marital status, residence, nationality, religion, monthly income *per capita* of family, type of medical insurance, diagnosis, phase of stroke, type of combined chronic disease, Brunnstrom stage, and main caregivers will be collected.

#### 2.6.2 Self-esteem

Self-esteem will be evaluated by the self-esteem scale with a single dimension and 10 items, which was compiled by Rosenberg in 1965 (29). Each item on the Chinese version of the self-efficacy scale is scored from 1 (quite wrong) to 4 (quite right). The total score ranges from 10 to 40 and a higher total score indicates a higher sense of self-efficacy. The Cronbach’s alpha of this scale is 0.83 (30), meaning good reliability.

#### 2.6.3 Rehabilitation self-efficacy

Rehabilitation self-efficacy will be measured by the Stroke Self-Efficacy Questionnaire compiled by Jones et al. in 2008, which is used to evaluate the functional performance and confidence of self-management of patients with stroke in the recovery period (31). Li et al. translated and revised it into Chinese with two dimensions, including activity of daily living efficiency and self-management efficiency (32). This scale has 11 items and each item is scored from 1 (very unconfident) to 10 (very confident). The total score ranges from 11 to 110, and a higher total score indicates a higher sense of rehabilitation self-efficacy. The Cronbach’s alpha of this scale is 0.969 (32).

#### 2.6.4 Positive emotion

The positive emotion dimension of the Positive Affect and Negative Affect Scale will be used to measure positive emotion in patients with stroke. This scale was developed by Watson et al. in 1988 and revised by Qiu et al. in 2008 (33). The positive emotion dimension has nine items and each item is scored from 1 (never) to 5 (almost). The total score ranges from 9 to 45, and a higher total score indicates more positive emotions. The Cronbach’s alpha of this scale is 0.92 (34).

#### 2.6.5 Upper limb function

Upper limb function will be measured by the Fugl-Meyer Assessment, which is one of the important scales for evaluating the motor function of stroke patients, with the advantages of reliability and high sensitivity (35). The upper limb dimension of the simplified Fugl-Meyer Assessment has 33 items. Each item is scored from 0 (cannot be completed) to 2 (completed). The total score ranges from 0 to 66. The higher the total score, the better the motor function of the upper limbs and hands.

#### 2.6.6 Activities of daily living

The modified Barthel Index will be used to evaluate the activities of daily living. This scale has 10 items, and each item is divided into 5 levels according to the degree of dependence (36). The total score ranges from 0 to 100. The higher the total score, the better the self-care ability.

#### 2.6.7 Safety outcomes and adverse events

All adverse events, such as pain, joint dislocation, and fracture, will be continuously measured during the rehabilitation period. Information about the severity, duration, and correlation to intervention of adverse events will be recorded in detail.

### 2.7 Statistical analysis plan

#### 2.7.1 Statistical methods

We will use IBM SPSS Statistics 22 to analyze the data, and all tests are two-sided. Statistical significance is defined as a *p*-value of <0.05. Confidence intervals (CIs) will be stated at the 95 confidence level. A P–P diagram will be used to test the normal distribution of measurement data. The statistical description of measurement data will use the mean and standard deviation when following a normal distribution; otherwise, use the median and quartile. When following normal distribution and homogeneity of variance, the measurement data will be compared by the analysis of variance for intergroup comparison, a paired-sample t-test will be used for intragroup comparison, and a multifactorial analysis of variance will be used to evaluate the interaction effects. Otherwise, the Kruskal–Wallis H-test will be adopted for intergroup comparison, and the Wilcoxon signed rank sum test will be used for intragroup comparison. Counting data will be described by number and percentage. The chi-square test and Fisher’s exact test will be used to analyze the counting data. If the baseline indicators are not comparable, an adjustment to the efficacy contrast of variables such as age, sex, and diagnosis is required. In patients where baseline indicators are unbalanced, an adjustment to the efficacy contrast of variables such as age, sex, and concurrent therapy is required. The covariance approach will be used for continuous variables, and logistic regression analysis will be used for counting data.

## 3 Discussion

This paper presents the study protocol of a randomized controlled trial, targeting older stroke patients with upper limb dysfunction. With this study, we will measure reminiscent music therapy combined with robot-assisted rehabilitation intervention and expect this intervention to promote the health status and self-care abilities of older patients with upper limb dysfunction after stroke. The novelty of reminiscent music therapy combined with robot-assisted rehabilitation lies in the combination of music therapy, reminiscence therapy, and robot-assisted rehabilitation. Given the fatigue and negative emotions that are easily generated by older stroke patients in the process of rehabilitation training, which affect their cooperation in rehabilitation treatment and hinder rehabilitation progress, we combine reminiscent music therapy with robot-assisted rehabilitation.

Neurological rehabilitation is of great significance for the prognosis of strokes. Upper limb dysfunction, especially in the hand, leads to more disabilities because its functions are more complex and vital to daily life (37). To deal with this problem, many approaches, including robot-assisted rehabilitation, have been suggested for upper limb and hand rehabilitation following stroke (38). The robot-assisted upper limb training is purposed to improve hand function and motor, sensory, and cognitive function by combining visual feedback with motivation (38). The clinical utility of robot-assisted rehabilitation has been increasing (39). Adding robot-assisted rehabilitation to usual rehabilitation could provide greater changes in the upper limb rehabilitation of stroke patients compared to usual rehabilitation alone (37). Reminiscence therapy has become one of the most feasible and effective social psychological interventions and can guide older people to review and re-experience past lives, which generates a new interpretation of life and helps older people to understand themselves, reduce the sense of loss, and increase self-esteem (40). Recalling happy events helps individuals maintain a positive self-image and improve self-esteem and happiness. On the contrary, recalling sad events helps individuals examine past situations so as to increase their ability to adapt to the existing environment and achieve self-integration (41, 42). Music is one of the most common triggers of nostalgia, and touching off memories with familiar songs could significantly improve the mental health of older people (24). Importantly, some studies suggest that the combination of music and rehabilitation will significantly improve the effect of rehabilitation training (26, 43).

Therefore, this study intends to combine reminiscent music therapy with robot-assisted rehabilitation in order to enable older stroke patients to carry out upper limb rehabilitation training under the stimulation of reminiscent music. To the authors’ knowledge, this is the first study to examine the effects of reminiscent music therapy combined with robot-assisted rehabilitation in older stroke patients with upper limb dysfunction. In our opinion, using reminiscent music to stimulate nostalgia may help stroke patients obtain a sense of perfection and satisfaction from memory and help them to maintain self-concept and enhance their sense of self-worth, so as to improve self-esteem, decrease negative emotions, and accept the conditions. It is conducive to improving rehabilitation self-efficacy, changing into a positive attitude toward disease, and reducing post-stroke fatigue. If the patient participates in the treatment and rehabilitation of the disease with a positive attitude and action, it will help to promote the recovery of the disease and improve the prognosis.

## Author contributions

QL: Conceptualization, Data curation, Investigation, Methodology, Software, Writing – original draft. ZL: Conceptualization, Methodology, Writing – review & editing. HC: Conceptualization, Methodology, Writing – review & editing. YX: Conceptualization, Methodology, Writing – review & editing. FW: Writing – review & editing. LL: Conceptualization, Methodology, Writing – review & editing. XH: Conceptualization, Funding acquisition, Methodology, Writing – review & editing.
